# Investigation Between the S377G3 *GATA-4* Polymorphism and Migraine

**DOI:** 10.2174/1874205X00802010035

**Published:** 2008-07-25

**Authors:** Chikhani Sherin, Fernandez Francesca, Poetter Karl, Toohey Brendan, Harvey Ron, Griffiths Lyn

**Affiliations:** 1 Genomics Research Centre, Griffith University, Gold Coast, Queensland, Australia; 2 Genera Biosystems Pty Ltd, Bundoora, Victoria, Australia; 3 The Victor Cardiac Research Institute, St. Vincents Hospital, Darlinghurst, Australia

**Keywords:** Migraine, genetic association, GATA 4 polymorphism, Heart disorder

## Abstract

Migraine is a common and painful neurological disorder, with genetic and environmental components. Several conditions have been shown to be comorbid with migraine, notably a cardiac malformation affecting the interatrial septum and leading to patent foramen ovale (PFO). Mutations in the development regulatory gene GATA-4, located on human chromosome 8p23.1-p22, have been found to be responsible for some cases of congenital heart defects including PFO. To determine whether the GATA-4 gene is involved in migraine, the present study performed an association analysis of a common GATA-4 variant that results in a change of amino acid (S377G), in a large case/control population (275 unrelated Caucasian migraineurs versus 275 control individuals). The results showed that there was no significant association for this polymorphism between migraine and controls (χ² = 0.84, P = 0.66). Thus it appears that the GATA-4 (S377G) mutation does not play a significant role in common migraine susceptibility.

## INTRODUCTION

Migraine is a common and debilitating neurological disease that affects a significant proportion of the population, usually affecting 10%-12% of the Western population [1] Characteristic manifestations of migraine include head pain, nausea, vomiting, photophobia and often severe neurological disturbances [2]. The most common forms of this disorder have been classified as migraine with aura (MA) and migraine without aura (MO) [2]. One of the most important aspects of the pathophysiology of migraine is the inherited nature of the disorder, with twin studies showing the importance of both genetic and environmental factors [3]. Migraine is now usually viewed as a polygenic multifactorial disease, with both environmental and genetic causative factors, and with multiple possibly interacting genes [4]. The age of onset for this disorder also varies, but in females it has been found that it is usually at puberty or shortly after, being less frequent in middle life and developing three times as often in woman as in men [5].

Several disorders have been shown to be comorbid with migraine, including epilepsy, asthma, depression, stroke and some congenital heart defects [6]. The cardiovascular association between migraine and ischaemic stroke is not coincidental and it could be due to a common genetic component and to the presence of different mutations in the same gene [7]. One heart disorder that has been associated with migraine is the cardiac malformation, patent foramen ovale (PFO) [6].

Patent foramen ovale results from an incomplete anatomic fusion of the atrial septum primum and secundum, which normally takes place shortly after birth [8]. This condition leads to a persistent connection between the right and left atrium of the heart, by which right-to-left shunt may result[8]. Large shunts more often than small shunts are associated with migraine with aura [9, 10]. PFO is believed to play a role in cryptogenic stroke *via *presumed paradoxical embolism [11]. The right to left shunt brought about by PFO could enhance migraine by affecting systemic levels of brain platelet neuromediators, like 5-hydroxytriptamine (5-HT), which is normally, inactivated by the pulmonary filter, thus triggering a migraine attack [12, 13].

Consequently, the increased risk of stroke in patients with MA could be explained by an increased propensity to paradoxical embolism [8]. The prevalence of PFO in the healthy population is approximately 20%-25%. A high prevalence of right to left shunt has been observed in patients with MA compared to healthy control subjects with a 41%-48% PFO prevalence in migraine with aura patients [11].

Migraine has been shown to be an independent risk factor for subsequent coronary heart disease events among women in the Women’s Health Study (WHS) and in men in the Physician’s Health Study (PHS) [11]. Recent evidence has shown that migraineurs who experience an aura are twice as likely to have a PFO compared to the general population [14]. Association between PFO and migraine has been found to be stronger in patients with MA than in those without aura [15]. Recent reports have emphasised an association between PFO, MA and stroke with 25 patients (PFO was considered to play a causal role in stroke) among 74 consecutive patients with cryptogenic stroke found to have a 52% prevalence of MA [12]. The incidence of PFO in patients with migraine is about 50% if migraine is accompanied by visual aura, versus 20% in the general population [16].

Mutations in developmental regulatory genes have been found to be responsible for some cases of congenital heart defects [17]. *GATA-4* is one such regulatory gene. It is part of the *GATA* family of zinc finger transcription factors [18], which play important roles in transducing nuclear events that modulate cell lineage differentiation during development and hypertrophy of adult cardiac myocytes [18].

To investigate the potential role of *GATA-4* gene in migraine an association study of the S377G polymorphism was conducted in a migraine case and age, sex and ethnicity matched control population. The S377G polymorphism (rs3729856) is located in exon 5 of *GATA-4* gene and was genotyped using a new silica bead-based technology. It is a non-synonymous coding polymorphism with an amino acid change (serine to glycine) and unpublished studies (R.H., L.G., unpublished data) have shown a reasonably high frequency of the S377G rarer allele in Caucasian populations (Australian Caucasians (n=400, 13.9%).

## MATERIALS AND METHODS

### Subjects

This research has been approved by the Griffith University Ethics Committee for experimentation on human subjects; all participants of the study gave consent. The association population has been matched for sex, age (+/- 5 years), and ethnicity. All subjects were of Australian Caucasian origin, interviewed by clinical neurologists and were diagnosed for either migraine without aura (MO) or migraine with aura (MA) according to the criteria of the International Headache Society [2]. None of the subjects were tested for PFO or other heart disorders. The study population comprised 275 migraineurs and 275 unrelated control individuals, with the control group matched for sex, age (+/- 5 years), and ethnicity, to minimize potential bias from population stratification.

### Genotyping

In this study we utilized standard polymerase chain reaction (PCR) analysis for the genotyping of the migraine case/control population (275 unrelated Caucasian migraineurs versus 275 control individuals), using forward primer: 5’ TGT CCC CGG CAA ATG TAG ATA AAG’3 and reverse primer: 5’ CAG TCG GCC TCC CCA CAA ACA GC ’3, resulting in a 318 base pair fragment following PCR. The thermocycler conditions were 94˚C for 15 minutes followed by 40 cycles at 94˚C for 30 seconds, 56˚C for 30 seconds, and 72˚C for 30 seconds. PCR was completed with an extended incubation of 5 minutes at 72˚C. The genotyping was undertaken by means of single nucleotide polymorphism (SNP) genotyping, using flow cytometry developed by Genera Biosystems. This is a silica bead-based SNP genotyping system that discriminates between alleles on the basis of competitive hybridisation between fluorescently labelled allele specific probes. In addition to the forward and reverse primers a mix containing three reagents specific for the SNP detection were used. These reagents consisted of, the AmpaSand^TM^ beads with a covalently bound oligonucleotides which is complementary to the ssDNA, an oligonucleotide probe specific for one allele labelled with a yellow or green dye and an oligonucleotide probe specific for the alternative allele labelled with a red dye. Each reaction was analysed by a FACSArray flow cytometer. Genotypes for all samples were determined by MPlots© software.

### Sequencing

The samples were sequenced using ABI Prism 377 dideoxy chain termination reaction. To confirm the results previously obtained by SNP genotyping, 5% of the population was sequenced. The sequences produced were analysed through the computer software CHROMAS.

### Statistics

Chi-square analysis was used to determine if the allele frequency of the S377G polymorphism in the *GATA-4* gene were associated or not with migraine, and if the allele frequencies were significant (α = 0.05). Chi-square analysis was also used to determine if the obtained genotype frequencies were in Hardy-Weinberg equilibrium.

### Ethical approval

This research was reviewed and approved by the Griffith University Human Research Ethics Committee (ethics protocol number MSC/05/05/HREC) and all subjects participating in the study gave informed consent.

## RESULTS

To determine whether the *GATA-4* mutation had an important role in migraine, an association analysis of the S377G polymorphism in a large case/control population of migraineurs was performed. Genotypes for 194 cases and 201 controls were determined for the case/control tested population (275/275). The genotype data can be observed in Table **1** as well as the allele frequency for the migraine and control populations. Statistical analysis of the S377G polymorphism revealed that there was no significant difference (χ² = 0.84, *P* = 0.66) between the migraine population and the controls. Moreover there was no significant difference observed in the migraine and control population with regards to female and males for this particular association study. Furthermore no association was found between migraine with aura and controls (χ² = 0.99, *P* = 0.61), nor between migraine without aura and controls (χ² = 0.2, *P* = 0.91). Hardy Weinberg equilibrium was investigated for both migraine cases and controls. It was found that allele frequencies did not deviate from Hardy Weinberg equilibrium in both the case and control groups (*P* = 0.91; *P* = 0.19).

## DISCUSSION

Six family members of have been identified in vertebrates to be part of the *GATA *family and have been subdivided in two groups: *GATA-1, GATA-2,* and *GATA-3*, which are expressed in the hematopoetic system [19]. *GATA-4, GATA-5* and *GATA-6*, which are critical for differentiation and cell-specific gene expression in different endoderm and mesoderm, derived tissues [19]. The human *GATA-4* gene is located in chromosome 8p23.1-p22 and contains 6 exons [20] and regulates its expression through its zinc finger [21]. It is widely expressed in the endoderm, mesoderm, heart, gonads, liver, small intestine and pancreas [19]. Functional studies have shown a role for the *GATA-4* gene in cardiac embryogenesis [21] and mutations in this gene have been found to be associated with congenital heart defects such as atrial septal defect (ASD) and patent foramen ovale (PFO) [17].

Reamon-Buettner *et al.* (2005) found six mutations in the N-finger of *GATA-4* in patients with PFO. This includes a homozygous deletion (677delC), that would lead to a frameshift mutation affecting critical residues arginine and histidine [21].

A number of studies have found a strong association between PFO and cryptogenic stroke, due to an increase of right heart pressure therefore predisposing to paradoxical embolism [22, 23]. A further association that has been increasingly reported is the one between migraine, particularly migraine with aura, and PFO [24]. A number of studies have shown an increased prevalence of PFO in young patients that present with migraine with aura and cryptogenic stroke [8, 16]. The severity of this disorder varies according to the size of the opening found in the heart of the patient. Family studies suggest that PFO is potentially associated with migraine with aura particularly as a risk factor for stroke in women [25, 26].

The *GATA-4* gene contains a common variation that results in a S377G polymorphism missense mutation with an alternative nucleotide of A/G, changing from the serine amino acid to the glycine amino acid. The variant is prevalent in a number of dispersed Caucasian populations and the allele frequency in Australian Caucasians is 13.9% (R.H. and L.G., unpublished data). It thus could conceivably play a role as a common contributing allele to a disorder like migraine. This study aimed to determine the role of the S377G polymorphism in the *GATA-4* gene in migraine and if there is a significant association between patients with migraine and this mutation. Genotypes were determined for the S377G polymorphism but results showed no association with migraine in the case/control groups studied. Moreover there was no significant association found between migraine with aura and the control population and no significant difference was observed between females with migraine versus control females for the migraine population.

Several studies have reported a higher prevalence of migraine in patients suffering from congenital heart disease [24, 26, 27]. Recently, Hirth and collaborators have studied the prevalence between congenital heart disease sufferers with migraine [28]. They reported an increased prevalence of migraine with aura in congenital heart disease more specifically in PFO patients (89% of these patients suffer from migraine) [28].

In addition, Tatdelide *et al*. has studied the prevalence of PFO in migraine sufferers [29]. They have found percentages of PFO in migraine patients with aura, without aura and the control group were 66.7%, 47.4% and 22.2%, respectively, results suggesting an association between PFO and migraine, especially with aura [29].

## CONCLUSIONS

Numerous studies have shown that mutations in the *GATA-4* gene have a significative role in cardiogenesis and are associated with congenital heart defects, like PFO. A previous mutation, homozygous deletion 677del C, has been found on the N-terminal zinc finger of *GATA-4* gene, and previously reported in PFO patients [28].

Our study has reported a lack of association of the S377G polymorphism in the studied migraine population, which does not support a correlation between this particular gene variant and migraine. It appears that the *GATA-4* (S377G) mutation does not play a significant role in common migraine susceptibility. However other variants in this gene may still play a role in migraine susceptibility. Further studies investigating other GATA-4 variants in families and case/control populations are warranted to more clearly determine whether this gene plays a role in migraine. Furthermore an association study investigating individuals that present with both PFO and migraine, particularly migraine with aura, would be very interesting and to this end we are currently collecting subjects with this pertinent information

## Figures and Tables

**Table 1:** Distribution of the S377G Polymorphism in Migraineurs and Controls of Original Sample (MO Migraine without Aura, MA Migraine with Aura)

Genotypes (n %)					Alleles (allele frequency %)		
Genotypes	AA	GA	GG	total (n)	A	G	total (n)
Migraine	158 (81%)	34 (18%)	2 (1%)	194	350 (90.2%)	38 (9.8%)	388
FEMALE	111 (81%)	25 (18.2%)	1 (0.8%)	137	247 (90%)	27 (10%)	274
MALE	47 (82.4%)	9 (15.8%)	1 (1.8%)	57	103 (90.4%)	11 (9.6%)	114
MA	93 (80.8%)	21 (18.31%)	1 (0.9%)	115	207 (90%)	23 (10%)	230
MO	65 (82.3%)	13 (16.5%)	1 (1.2%)	79	143 (90.5%)	15 (9.5%)	158
CONTROL	169 (84.1%)	29 (14.4%)	3 (1.5%)	201	367 (91.3%)	35 (8.7%)	402
FEMALE	122 (84.1%)	20 (13.8%)	3 (2.1%)	145	264 (91%)	26 (9%)	290
MALE	47 (83.9%)	9 (16.1%)	0 (0)	56	103 (92%)	9 (8%)	112

**Table 2:** Chi-Squared (χ^2^) Analysis of all Migraine Groups Against Controls for the S377G Polymorphism

	Genotypes		Alleles	
	χ^²^	P-value	χ^²^	P-value
Migraine vs Control	0.84	0.66	0.28	0.6
Subtypes	χ^²^	P-value	χ^²^	P-value
MA vs Control	0.99	0.61	0.29	0.59
MO vs Control	0.2	0.91	0.09	0.77
Mig Female vs Control Female	1.85	0.4	0.13	0.72
Mig Male vs Control Male	0.99	0.61	0.18	0.67
